# Telomere-to-telomere and haplotype-resolved genome of the kiwifruit *Actinidia eriantha*

**DOI:** 10.1186/s43897-023-00052-5

**Published:** 2023-02-17

**Authors:** Yingzhen Wang, Minhui Dong, Ying Wu, Feng Zhang, Wangmei Ren, Yunzhi Lin, Qinyao Chen, Sijia Zhang, Junyang Yue, Yongsheng Liu

**Affiliations:** 1 School of Horticulture, Anhui Agricultural University, Hefei, 230036 China; 2 School of Forestry Science and Technology, Lishui Vocational and Technical College, Lishui, 323000 China; 3 Ministry of Education Key Laboratory for Bio-Resource and Eco-Environment, College of Life Science, State Key Laboratory of Hydraulics and Mountain River Engineering, Sichuan University, Chengdu, 610064 China

**Keywords:** Kiwifruit, *Actinidia eriantha*, Genome, Telomere-to-telomere, Haplotype, Centromeric

## Abstract

*Actinidia eriantha* is a characteristic fruit tree featuring with great potential for its abundant vitamin C and strong disease resistance. It has been used in a wide range of breeding programs and functional genomics studies. Previously published genome assemblies of *A. eriantha* are quite fragmented and not highly contiguous. Using multiple sequencing strategies, we get the haplotype-resolved and gap-free genomes of an elite breeding line “Midao 31” (MD), termed MDHAPA and MDHAPB. The new assemblies anchored to 29 pseudochromosome pairs with a length of 619.3 Mb and 611.7 Mb, as well as resolved 27 and 28 gap-close chromosomes in a telomere-to-telomere (T2T) manner. Based on the haplotype-resolved genome, we found that most alleles experienced purifying selection and coordinately expressed. Owing to the high continuity of assemblies, we defined the centromeric regions of *A. eriantha*, and identified the major repeating monomer, which is designated as *Ae-CEN153*. This resource lays a solid foundation for further functional genomics study and horticultural traits improvement in kiwifruit.

**Graphical Abstract:**

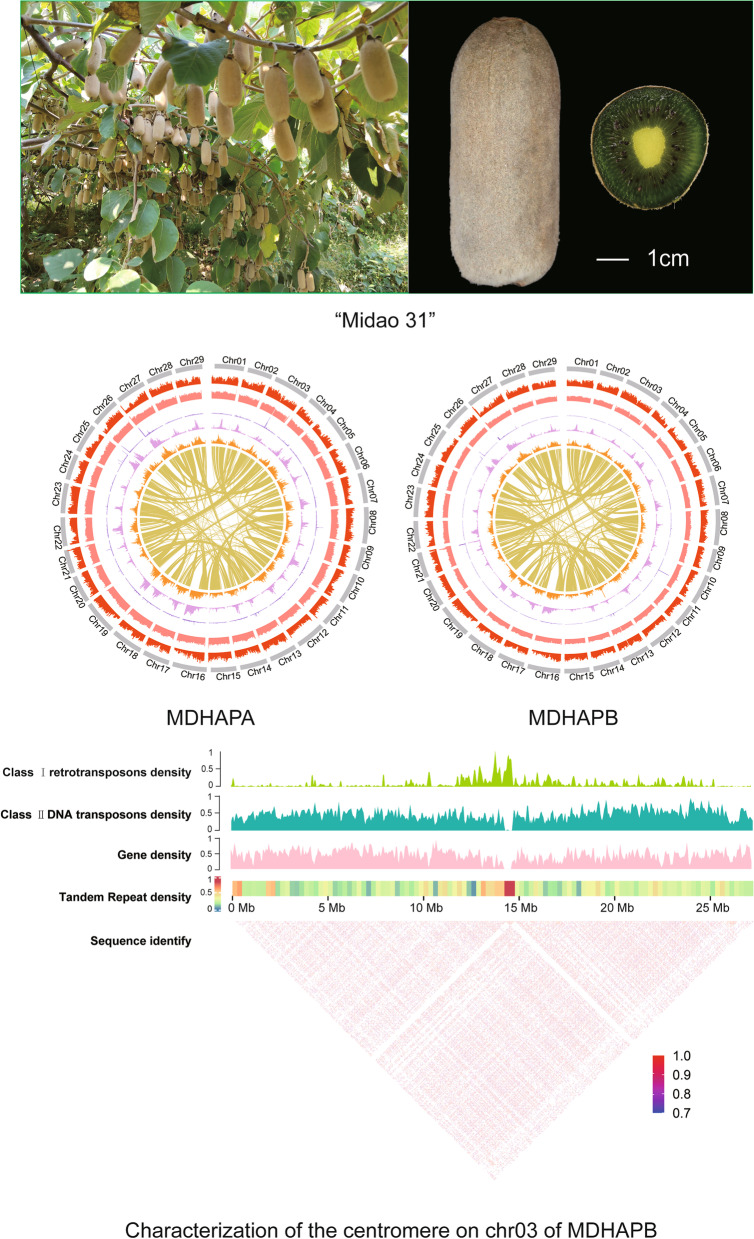

**Supplementary Information:**

The online version contains supplementary material available at 10.1186/s43897-023-00052-5.

## Core

The telomere-to-telomere and haplotype-resolved genome of *A. eriantha* filled most gaps and greatly improved the assembled genome quality, for the first time revealing the structure of centromeres and telomeres, laying the foundation for a better understanding of the structure and function of the kiwifruit genome.

## Gene and accession numbers

The genome sequencing data and transcriptome sequencing data have been deposited at Sequence Read Archive database in NCBI and the accession numbers is PRJNA905539.

## Introduction

Since the first draft genome of “Hongyang” kiwifruit was published in 2013 (Huang et al. 2013), several genomes have also been released, including *A. chinensis* (Pilkington et al., 2018, (Wu et al. 2019*), A. eriantha* (Tang et al., 2019, (Yao et al. 2022*),* which provided a valuable resource for facilitating kiwifruit breeding and studies of kiwifruit biology. However, due to the limitations of past technologies, these genomes still contain a large number of gaps, leaving centromeres, telomeres, and other highly repetitive regions unfinished, even though these regions contain a lot of important information. Telomeres are highly repetitive DNA sequences at the ends of chromosomes that protect them from degeneration during cell division (Shay et al. 2019). It is essential for the structure and stability of chromosomes and involves important biological processes, such as flowering time (Choi et al. 2021) and aging (Chakravarti et al. 2021). In addition, the centromere is another distinct chromosomal domain that serves as the docking site for the assembly of the kinetochore for chromosome segregation (Wu et al. 2011). Plant centromeric DNA is mainly composed of tandem repeats, retrotransposons, and low-copy sequences. A recent study showed that few active genes exist in the centromeric region (Ma et al. 2007). With the development of sequencing technology, both ultra-long Oxford Nanopore Technologies (ONT) and Pacific Biosciences (PacBio) high-fidelity (HiFi) data were used to resolve the gaps in plant and animal genomes. Using the third-generation DNA sequencing technique in combination with second-generation Hi-C data, it is possible to completely assemble an accurate, gap-free genome of kiwifruit.

Recently, the T2T genome of *A. chinensis* (Hongyang v4.0) have been released (Yue et al. 2022), which filled most gaps in the previously released genome, and reveals the structural characteristics of telomeres and centromeres in kiwifruit for the first time. As another important cultivated species in the *Actinidia* genus, *A. eriantha* has drawn the attention of scientists in recent years, because of its higher content of vitamins, s horizontal disease resistance, high efficiency in genetic transformation, and relatively short juvenile phase (Wang et al. 2006). A series of new cultivars, such as “White” (Wu et al. 2009) and “Ganmi 6” (Xu et al., 2015), have been bred, greatly enriching the varieties of kiwifruit. Although two versions of the *A. eriantha* genomes were previously published, they still contained numerous gaps and assembly errors. Neither of the two previous versions of the *A. eriantha* genome reached T2T levels, limiting our understanding of the structure and function of *A. eriantha* genomes.

Here, we reported a gap-free genome of *A. eriantha* by using an elite breeding line (namely Midao 31) derived from the hybrid progenies between “White” and “MHX-1”, which has the advantages of large fruit and high yield (Supplementary Fig. 1). The genome was assembled with high-coverage and accurate long read sequence data using multiple assembly strategies. For the first time, this version of the genome reveals the structure of highly repetitive regions of *A. eriantha* such as centromeres and telomeres, laying the groundwork for a better understanding of the structure and function of the kiwifruit genome.

## Results

### Genome sequencing and assembly

In order to obtain the T2T *A. eriantha* genome, we introduced multiple sequencing technologies to generate high-quality sequences (Supplementary Table 1). PacBio sequencing yielded 29.7 Gb of HiFi clean reads (~ 49 X genome coverages) with an N50 of 17.1 Kb; Oxford Nanopore Technologies yielded 20.6 Gb (~ 34X genome coverages) with an N50 of 100.4 Kb; A total of 105.5 Gb of clean reads (~ 175X genome coverages) were generated from Hi-C libraries; The Illumina short-reads data (~ 8X genome coverages) from “Midao31” were used to polish ONT long-reads.

First, the HiFi reads were used for primary assembly of *A. eriantha* by hifiasm, generating two preliminary assemblies with a size of 633 Mb and 622 Mb. The two preliminary assemblies include 237 contigs with the N50 length of 21 Mb and 185 contigs with the N50 length of 18 Mb respectively, which are greatly larger than the previous two versions of *A. eriantha* (Table 1). Then the contigs of two haplotypes were anchored to 29 scaffolds by juicer (Durand et al. 2016) and 3D de novo assembly (3D-DNA) pipeline (Dudchenko et al. 2017) using Hi-C reads. It was worth noting that the 29 scaffolds still contain a lot of gaps and incorrect joins because the HiFi contigs were optionally split in regions lacking Hi-C coverage. Then we re-mapped the HiFi contigs of two haplotypes against the corresponding 29 Hi-C scaffolds for de novo genome assembly with a reference-guided strategy by using a custom Perl script (https://github.com/aaranyue/CTGA). We consequently obtained two preliminary chromosome-level genomes that contain 8 gaps and 17 gaps, respectively. Meanwhile, the polished ONT reads were used to fill gaps using the TGS-GapCloser (Xu et al., 2019). Finally, we get two final haplotype *A. eriantha* genomes, termed MDHAPA and MDHAPB, with a size of 619.3 Mb and 611.7 Mb, respectively, including 27 and 28 gap-close chromosomes (Table 1). The remaining three gaps were distributed on chr13, chr21 of MDHAPA, and chr21 of MDHAPB (Supplementary Table 2). Our two haplotype assemblies, with greatly improved contiguity and completeness, showed high synteny with the four published genomes of “White”, “wild”, “Hongyangv4.0” and “Red5” (Supplementary Fig. 2).

**Table 1:** Summary of genome assembly and annotation of* A. eriantha*

**Genomic feature**	**MDHAPA**	**MDHAPB**	**White**	**Wild**
Total size of assembled contigs (Mb)	633	622	690.4	655
Number of contigs	237	185	4076	/
N50 value of contigs length (Mb)	21	18	0.54	2
Total size of assembled genomes (Mb)	619.3	611.7	690.6	657.1
Number of gaps	2	1	2341	709
Number of gap-close chromosomes	27	28	0	0
Number of telomeres (pairs)	24	25	/	/
Number of definite centromeres	29	29	/	/
TE size (%)	40.92	41.11	43.32	41.29
GC content (%)	35.67	35.68	/	/
Genome BUSCOs (%)	99	99.2	93.8	93.2
LTR assembly index score	21.52	20.45	/	/
Number of genes	46,008	47,184	42,988	41,521
Gene BUSCOs (%)	92	91.5	/	/
QV	59.60	50.93	/	/

### Genome annotation and assembly assessment

Within the two haplotype assemblies, 253.46 Mb (40.92%) and 251.48 Mb (41.11%) of repetitive sequences were found (Table 1), similar to those in the genomes of “White” (43.32%) and “wild” (41.29%). Among them, 25.45% and 24.67% were long terminal repeats (LTRs), and 15.24% and 12.45% were terminal inverted repeats (TIRs) in MDHAPA and MDHAPB, respectively (Supplementary Table 3). We predicted a total of 46,008 and 47,184 high-confidence protein-coding genes from MDHAPA and MDHAPB, respectively, which were close to the 45,809 genes predicted in the HY4P genome and the 45,434 predicted in the HY4A genome (Yue et al. 2022) and showing 93.8% and 93.2% BUSCO completeness (Table 1). The total lengths of the MDHAPA and MDHAPB genes were 249.73 Mb and 252.05 Mb, respectively. Of these protein-coding genes, 39,836 (86.58%) and 40,328 (85.47%) could be functionally annotated to a comprehensive database of eggNOG. Compared to the “White” genome, more than 4000 genes were newly predicted in the two assemblies. And a large number of genomic variations between MDHAPs and White were discovered to be associated with these new predicted genes (Supplementary Table 4). KEGG analysis revealed that these genes functionally enrich in multiple pathways, including RNA degradation, plant-pathogen interaction, pentose glycosylation, and so on (Supplementary Fig. 3a and 4a). GO analysis showed that the newly predicted genes were mainly enriched in the biological process and molecular function categories (Supplementary Fig. 3b and Fig. 4b). Among them, a lot of genes were related to cold acclimation and GTPase activity. So, these newly predicted genes may involve important biological functions in kiwifruit.

**Fig. 1:**
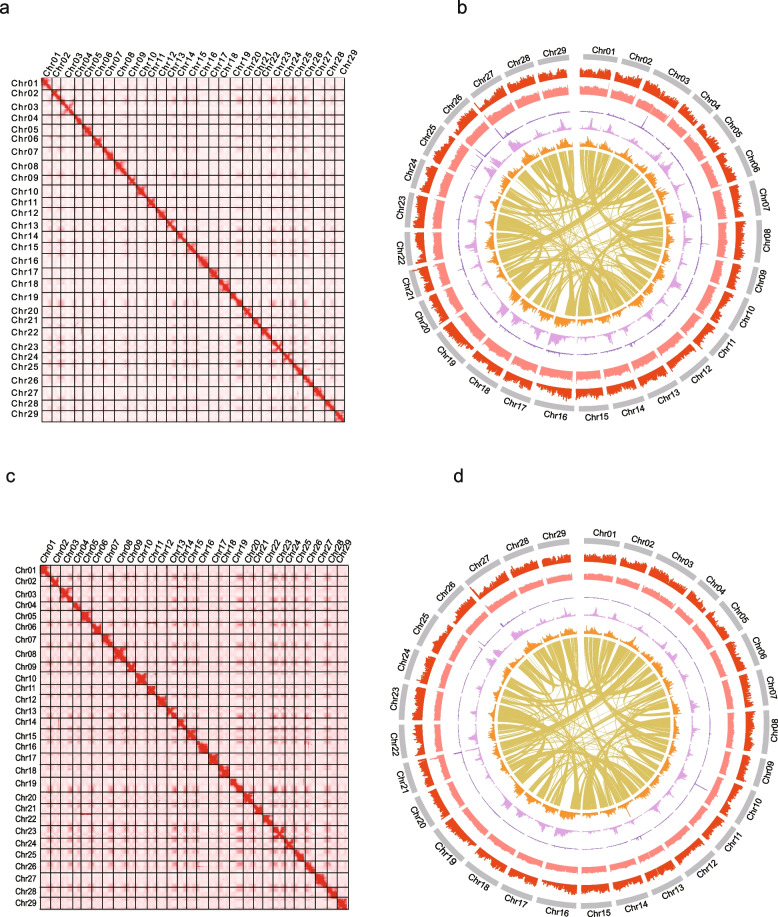
Hi−C map and overview of the genomic features of of *A. eriantha*. Heatmap showing Hi−C interactions of MDHAPA (**a**) and MDHAPB (**c**); The genomic features of MDHAPA (**b**) and MDHAPB (**d**). Tracks from outside to inside are chromosomes number, gene density, GC content, tandem repeat density, LTR/Gypsy density, LTR/Copia density, links between genepair

Multiple evaluative approaches were used to confirm the high quality of haplotype assemblies: (i) The assembly spectrum copy number plots plotted by the KAT program (Mapleson et al. 2017) clearly demonstrated that the phasing of the assembled haplotypes is correct (Supplementary Fig. 5), (ii) The 97.25% Illumina reads, the 99.49% HiFi reads, and the 99.99% were successfully aligned to the two assemblies (Supplementary Table 5), suggesting that the *A. eriantha* genome was sufficiently covered by the assembly; (iii) Hi-C interaction matrices displayed a diagonal pattern for the intra-chromosomal interactions in all pseudochromosomes, indicating the right ordering and orientation (Fig. 1b and 1d); (iv) A total of 1597 (99.0%) and 1601 (99.2%) complete BUSCO genes were identified in the two haplotype genomes, respectively, which was superior to the genome of the “White” and “wild” (Supplementary Fig. 6); (v) The high LAI scores ( 21.52 and 20.45) were observed in two haplotype genomes (Table 1). (vi) The consensus quality value (QV) of MDHAPs was 59.60 and 50.93, indicating the high accuracy of our assemblies (Table 1).

### Haplotypic variations and spatiotemporal expression pattern of alleles

**Fig. 2:**
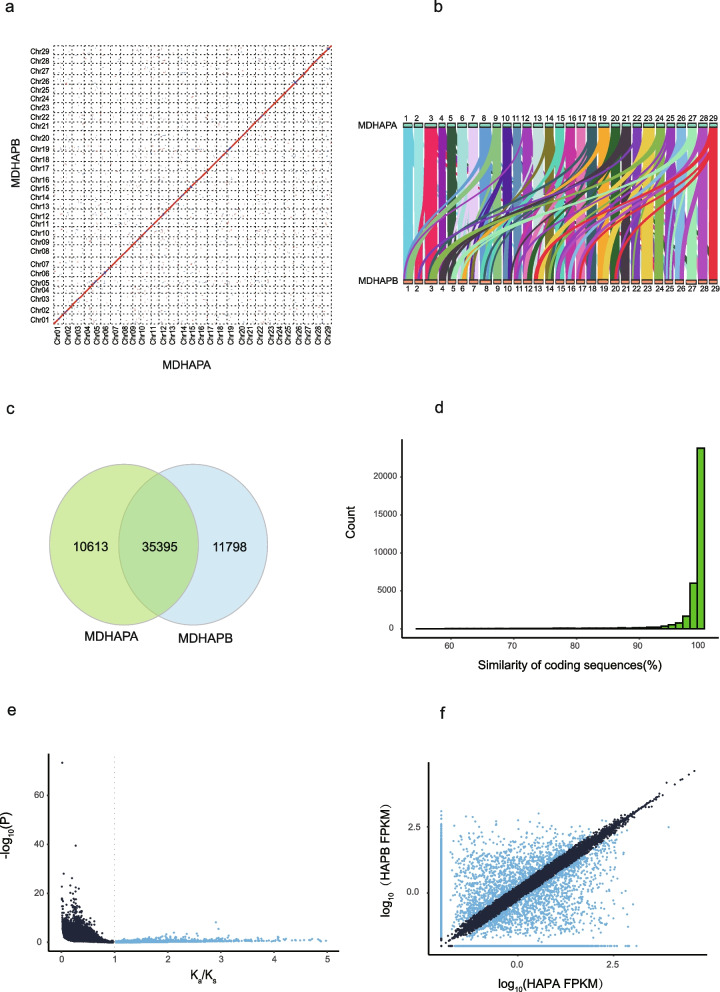
Comparison of MDHAPA and MDHAPB. **a** Sequence alignment of MDHAPA and MDHAPB. **b** The syntenic regions between MDHAPA and MDHAPB. **c** The number of alleles found whithin genome−wide alignmentblocks. **d** Similarity of coding sequences for alleles. **e** Pairwise comparison of the Ka/Ks distribution for allelic genes. **f** Identification of ASE genes in fruit of DAF 120. Coordinates are logarithmically scaled (log10). Blue dots indicate ASE genes, and gray dots represent genes that are not ASE genes

Syntenic analysis revealed highly consistent sequence orders between the two haplotypes (Fig. 2a). Genomic collinearity analysis between MDHAPA and MDHAPB revealed that 45,295 transcripts matched 540 syntenic blocks (Fig. 2b). We also identified substantial variations between the two haplotypes by using SyRI, including 2,574,323 SNPs, 457,407 insertions/deletions, 289 inversions, and 167 translocations (Supplementary Table 6). These variations spanned approximately 35.7 Mb, representing 5.6% and 5.7% of the assembled MDHAPA and MDHAPB genomes, respectively.

Correlated or differential expression of alleles could have profound effects on growth and evolution. Using MCscanX, we separated 35,395 pairs of alleles in the haplotype-resolved genome (Fig. 2c). These alleles are relatively evenly distributed across the 29 chromosomes of kiwifruit. Most allelic genes maintained high levels of coding sequence similarity (mean = 98.13%) (Fig. 2d). To evaluate the natural selection pattern of alleles, we calculated the K_a_/K_s_ value between allelic gene pairs. The results revealed that the majority of allelic genes had clearly experienced purifying selection (K_a_/K_s_ < 1), with only a small number of allelic pairs showing possible positive selection (K_a_/K_s_ > 1) (Fig. 2e).

Then we investigated the allele-specific expression (ASE) across different tissues (fruit at different development stages, leaf and stem). A total of 2904 allelic genes showed different expression patterns (log2 fold change > 1, p value < 0.05) (Fig. 2f), while others did not show a more than two-fold difference in expression, indicating that most alleles in the *A. eriantha* genome were coordinately expressed. These genes showed functional enrichment in biological processes such as regulation of lipids, activation of protein kinase activity, and cellular components including polysomal and vesicle tethering complexes based on the gene ontology (GO) annotation (Supplementary Fig. 7a). The result of KEGG pathway annotation revealed that the ASEs had functional enrichment in multiple biological processes, including endocytosis, cellular senescence, and circadian rhythm (Supplementary Fig. 7b), suggesting that a potential mechanism to overcome deleterious mutations occurred in important genes related to basic biological functions.

Among the 2904 ASEs, 786 genes showed expression biased toward one allele across six samples, which were defined as consistent ASE genes (Supplementary Fig. 8a). Genes with expression biased toward one parental allele in some samples but shifted to another allele in other samples were defined as inconsistent ASE genes, which indicate an overdominance effect. A total of 103 inconsistent ASE genes were found in the *A. eriantha* “Midao31” genome (Supplementary Fig. 8b), which was less than the number of consistent ASE genes. Then we investigated the functional impact of the allelic variations and found that 29.97% of SNPs and 30.88% of InDels caused changes in the upstream sequence (+ 2 kb) (Supplementary Table 7), indicating that the promoter sequence variation may be an important cause of allelic differential expression.

### Detection of the telomere and centromere locations at chromosomes

Telomeres are nucleoprotein structures at the ends of chromosomes and function to maintain genome stability and consist of a tandem repeat of TG-rich microsatellite sequence (Turner et al. 2019). The telomeric repeat sequenece is TTTAGGG in most plants (Fajkus et al. 2005). Using the seven-base telomeric repeat (CCCATTT at the 5' end and TTTAGGG at the 3' end) as a query, we identified 54 telomeres, resulting in 25 T2T pseudomolecules both in MDHAPA and MDHAPB assemblies (Supplementary Table 8 and Supplementary Table 9). The total counts of telomere repeats ranged in 176 ~ 3140 and 111 ~ 2760, with a mean value of 1318 and 1063.

**Fig. 3:**
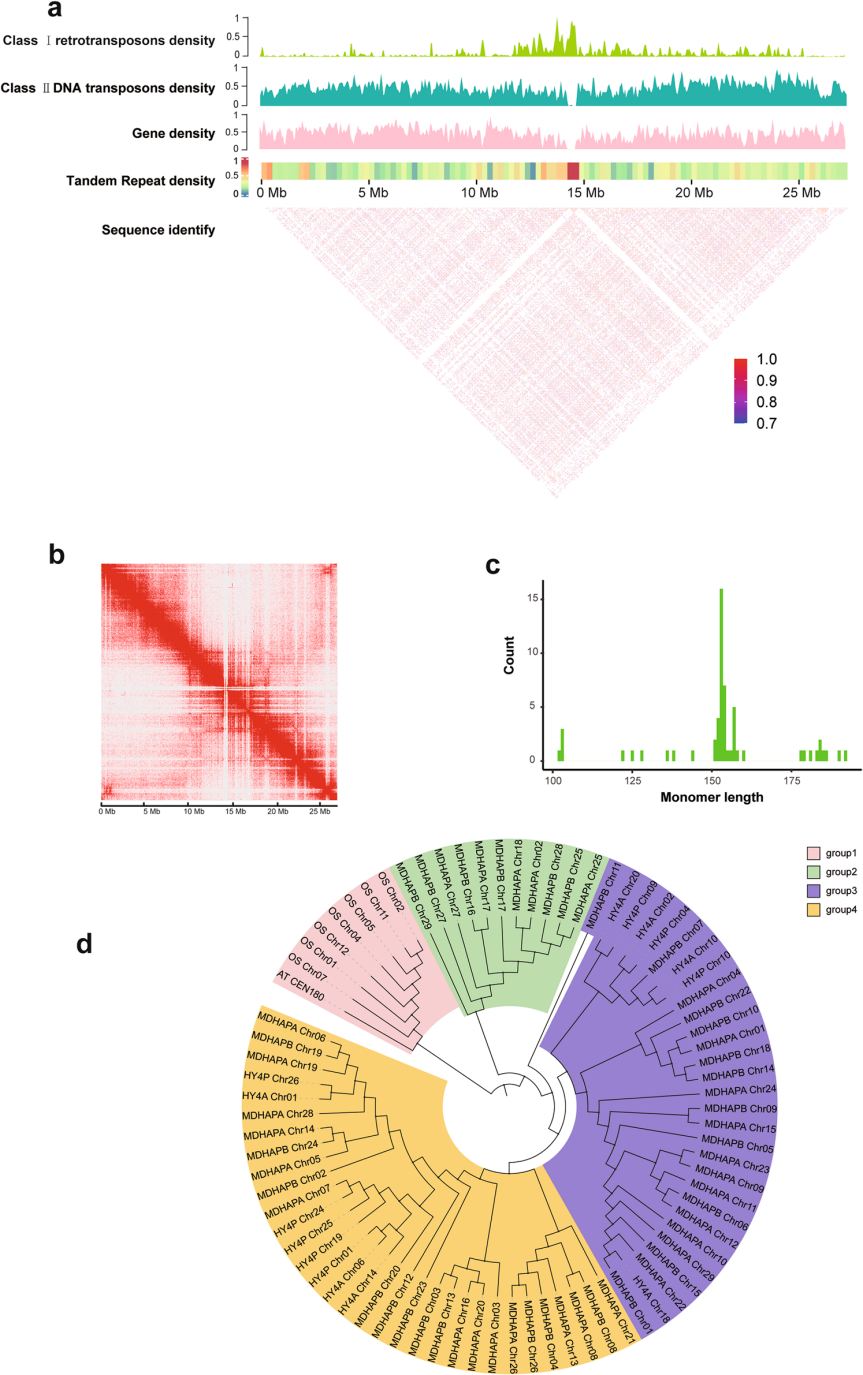
Characterization of the *A.eriantha* centromere. **a** Characterization of the centromere on chr03 of MDHAPB. The heatmap showing pairwise similarity of 50 Kb sequence along the whole chromosome of Chr03. **b** Heatmap showing Hi−C interactions of the chromosome03 of MDHAPB. **c** The length distribution of major monomer in *A. eriantha*. **d** The phylogenetic tree of centromeric monomers from *A. eriantha*, *A. chinensis*, *Arabidopsis* and rice

The centromere is an important part of chromosomes and plays a crucial role in the proper segregation of chromosomes. Unfortunately, information about the centromeres of kiwifruit is limited. One of the main reasons is that the centromere region contains highly repetitive sequences, which impede assembly from short DNA sequencing reads (Nurk et al. 2022). Nevertheless, the development of sequencing technology allow us to have the opportunity to assemble centromere regions. To identify the location and sequence features, we used the Tandem Repeats Finder (TRF) tool to search tandem repeats in our assemblies, and only the repeat monomers with lengths ranging from 100 to 200 bp were retained. And then CD-HIT (Fu et al. 2012) was used for clustering these monomers to reduce sequence redundancy and improve the precision of centromere localization based on sequence similarity search, the continuous and high-frequency regions were thought to be approximate centromeric sequences. Finally, we determined the location of the centromeres of all chromosomes in two haplotype assemblies. The result showed that the centromere boundaries of the two haplotype genomes had similar positions on the chromosome, and the length of the centromere region ranged from 217,369 bp to 1,893,971 bp in MDHAPA and from 112,182 bp to 1,168,845 bp in MDHAPB (Supplementary Table 10). Aside from that, there are 147 and 151 new genes predicted in the centromere region of MDHAPA and MDHAPB, respectively (Supplementary Table 11 and 12). To verify the accuracy of the centromere region, we analyzed the gene density, repeat distribution, and sequence similarity on the chromosome (Fig. 3a). The distribution of repeats revealed that the class I retrotransposons are more common in centromeres, while the class II retrotransposons were more evenly distributed across the genome, which was similar to other species such as *Brassica* (Perumal et al. 2020) and *A. chinensis* (Yue et al. 2022). In addition, the centromere region has low gene density and low similarity compared with other regions on the chromosome (Fig. 3a). Finally, the Hi-C heatmap also showed that the location of centromere region was correct(Fig. 3b).

Based on the analysis of tandem repeats in the centromere region, we found that the repetition types in the centromere region of kiwifruit are quite complex, containing one major repeating monomer and several minor repeating monomers. The length of the major repeating monomer ranged from 102 to 192 bp (Supplementary Table 13 and 14), and most of the major repeating monomer’s length was 153 bp (named *Ae-CEN153*) (Fig. 3c), which was consistent with *A. chinensis*. Alignment of the *Ach-CEN153* and *Ae-CEN153* sequences from *A. chinensis* and *A. eriantha*. Alignment of the *Ach-CEN153* and *Ae-CEN153* sequences from *A. chinensis* and *A. eriantha* revealed that they retain strong similarities (Supplementary Fig. 9). These results indicated that the centromere repeat unit sequences were relatively conserved in the two species. The phylogenetic analysis of the representative monomers of rice, *Arabidopsis, A. chinensis* and all monomers of *A. eriantha* revealed that all monomers could divided into four groups, the kiwifruit monomers were separated from *Arabidopsis* and rice (Fig. 3d), implying that the kiwifruit centromere tandem repeats have little homology with rice and *Arabidopsis*.

## Discussion

Over the past few years, the complete telomere to telomere (T2T) assembly of *Arabidopsis* (Naish et al., 2021), rice (Song et al. 2021), banana (Belser et al. 2021), and watermelon (Deng et al. 2022) has been reported. As an important fruit crop worldwide, kiwifruit plays an important role in the world agricultural economy. However, the genome of kiwifruit has remained fragmented and contains a lot of gaps, which impede functional genomics and genetic improvement in kiwifruit. As an important resource for kiwifruit breeding and genetic studies, *A. eriantha* was well known for its remarkable vitamin C content and great nutritional value. In this study, we successfully assembled a T2T gap-free genome using a combination of multiple sequencing platforms. The PacBio HiFi sequencing provided the highly accurate reads and generated two high quality assemblies with contig N50 sizes of 21 Mb and 18 Mb, respectively. The ONT sequencing provides long reads (N50 > 100 kb), which contribute to the assembly of highly repetitive regions. Using Hi-C scaffolding, short-read polishing, and manual curation, we elevated the reference genome of *A. eriantha* to a higher quality level. Thanks to the highly continuous genome, we have predicted more genes than “White” and “wild”, and a lot of these extra genes correlate with some important biological processes. The result showed the integrity and accuracy of two haplotypes were superior to other *A. eriantha* reference genomes.

As we all know, parental alleles are randomly selected or collapsed during genome assembly (Shi et al. 2019), and those genomes with high levels of heterozygosity potentially, such as the kiwifruit genome, contain many mosaic sequences. Haplotype assemblies can be a good solution to this problem and facilitate genetic studies, especially in linkage analysis, population genetics, and functional studies. In this study, the long-read sequencing technology and haplotype assembly techniques were adopted to achieve two haplotype assemblies. Due to the mosaic sequences being split into different haploid genomes, the size of the two haplotype genomes was lower than the “White” genome. We also find abundant structural variations between the two haplotypes, these variations may be a major contributor to genetic diversity and adaptive evolution. The information about ASE in the kiwifruit genome was limited, because the haplotype-resolved genomes are lacking. In this study, we found that most ASEs tend toward consistently expressing, but only a small number of ASEs displayed consistent expression, indicating that the dominant effect may play a major role in the *A. eriantha* genome. Based on the new haplotype genomes, we first defined the locations of centromeres in *A. eriantha*. Although centromeric regions are highly repetitive and have a relatively low gene density as compared to other parts of the chromosome, there are still some new genes found in these regions. We found that the repeat type of centromeric regions in kiwifruit was relatively complex, but the main centromeric monomer in *A. eriantha* and *A. chinensis* was relatively conservative. These results laid the foundation for the sequence identity and functional analysis of centromere regions in kiwifruit.

In conclusion, we present a high-quality haplotype-resolved reference genome of *A. eriantha* in this study, and it provides useful resources for the comparative genomics, molecular biology, molecular breeding, genetics, and evolutionary studies of kiwifruit.

## Methods

### Plant materials and sequencing

“Midao 31”, a hybrid between *A. eriantha “*White” (female) and *A. eriantha* “MHX-1” (male), was used in this study. High-quality genomic DNA was extracted from fresh young leaf tissue from “Midao 31”, growing in Hefei, Anhui Province, China, and separately packaged for PacBio HiFi, ONT ultra-long, and Hi-C sequencing. Tissue materials from leaves, stems, and fruits were used for RNA-seq and genomic annotation.

The kiwifruit genomic DNA (Allen et al. 2006) was prepared using a modified CTAB method and evaluated using an Agilent 2100 Bioanalyzer (Agilent Technologies, Santa Clara, CA, USA) and Qubit fluorometer instrument (Thermo Fisher Scientific, MA, USA). The library for ONT sequencing was constructed using the 1D ligation sequencing kit (SQK-LSK108, ONT, UK) and sequenced on the ONT PromethION platform. The Hi-C libraries were then constructed as a standard procedure, including chromatin extraction and digestion, DNA ligation, and purification. For PacBio HiFi sequencing, a standard SMRTbell library was prepared with 50 μg of gDNA by using the SMRTbell Express Template Prep Kit 2.0, according to the manufacturer’s instructions. SMRTbell libraries were then sequenced on a PacBio Sequel II system (Pacific Biosciences, CA, USA).

### Genome assembly and assessment

The HiFi reads were de novo assembled into two haplotype contigs using the hifiasm v0.16.1 software with default parameters (Cheng et al. 2021). And then the two haplotype contigs were corrected, grouped, sorted, and anchored to pseudochromosomes with Hi-C reads using juicer (Durand et al. 2016) and 3D-DNA software (Dudchenko et al. 2017). Then, a custom Perl script was used to remap the HiFi contigs against the Hi-C pseudochromosomes using a reference-guided strategy (https://github.com/aaranyue/CTGA). The ONT reads were polished by Pilon (Walker et al., 2014) and then used to fill gaps in the ref-guided pseudochromosomes by the TGS-GapCloser (Xu et al., 2019). To evaluate the reliability of our assembly, the HiFi reads and the Illumina reads were remapped to the two assembled haplotypes genomes using minimap2 (Li et al., 2018). The completeness of two assemblies was evaluated by mapping the Benchmarking Universal Single-Copy Orthologs (BUSCO) to the genomes using BUSCO v3.0.2 (Manni et al. 2021) with the Embryophyta odb10 dataset. The continuity was assessed using the LTR Assembly Index (LAI) (Ou et al., 2018). The consensus quality (QV) value was assessed using Mequery (v1.3) (Rhie et al. 2020).

### Gene and repeat annotations

Gene structure annotation was performed by the braker software (Hoff et al. 2019) using a combination of de novo prediction and transcript evidence from ten RNA-seq datasets (Supplementary Table 15). Only genes that met the criteria of having start and stop codons and being longer than 100 nucleotides were reserved. Gene function was annotated by eggmapper against a series of protein sequence databases. GO and KEGG enrichment analyses were performed using the R package clusterProfiler (Yu, 2012). Transposable elements (TEs) in the *A. eriantha* genome were identified using the Extensive de novo TE Annotator (EDTA) with default parameters (Ou et al. 2019). The tandem repeats (TRs) were identified by the TRF software (Benson 1999) with parameters (2 7 7 80 10 50 500 -f -d -m).

### Genome comparison and synteny analysis

The genomic sequences of “White” and “wild” were downloaded from the KGD database (http://kiwifruitgenome.org/) (Yue et al. 2020) and the NCBI database. The MUMmer software (Marcais et al., 2018) was used for the alignment comparison analysis between MDHAPA, MDHAPB, and the “White” and “wild” genomes with parameters (–maxmatch -c 500 -b 200 -l 100). The alignment was filtered using the delta-filter implemented in Mummer with the parameters (-m -i 90 -l 2000). Then, the filtered result was visualized by mummerplot. The collinearity, structural variations, and sequence differences analysis was performed between MDHAPA and MDHAPB using the Synteny and Rearrangement Identifier (SyRI) (Goel et al. 2019).

### Allelic gene identification and expression analysis

MCScanX (Wang et al., 2012) was used to identify synteny blocks between a pair of allelic chromosomes, paired genes within each synteny block with high similarity were considered alleles A and B. The K_a_/K_s_ value was calculated using KaKs_Calculator V2.0 (Wang et al., 2010). The RNA-seq data from 6 samples (fruit DAF 20, fruit DAF 40, fruit DAF 60, fruit DAF 120, leaf, and stem) were aligned to the MDHAPA and MDHAPB using HISAT2 v2.0.0 (Kim et al. 2015). The FPKM values were estimated using featureCounts v1.5.3 (Liao et al. 2014). The differentially expressed genes were identified using DESeq2 (Love MI et al., 2014) and edgeR (Robinson MD et al., 2010). ASE was determined if the log_2_(fold change) values of FPKM between two alleles were greater than 1 and the P value < 0.05. The ASE patterns (consistent and inconsistent expression pattern) was identified using the same methods as the tea plant *Camellia sinensis* genomes project (Zhang et al. 2021).

### Telomere detection and centromere localization

The telomere was identified by the Telomere Identification toolKit (tidk) using the normalized and unified sequence “AAACCCT” for search. A pipline containing TRF tools, and CD-HIT tools was used for the identification of the centromere region. The TRF tools were used to identify whole-genome tandem repeats and monomers. The CD-HIT was used to cluster these monomers to reduce sequence redundancy. Those continuous and high-frequency regions were regarded as candidate centromere regions. At last, combine the result of gene density, TE number with candidate regions to predict the most likely centromere location.

### Phylogenetic tree construction and sequence alignment of centromeric monomers

The representative centromeric monomer sequences of *Arabidopsis* (Wang et al. 2022), rice (Song et al. 2021), *A. chinensis* and *A. eriantha* were then aligned by muscle (Edgar 2004), and phylogenetic analysis was performed by the Maximum Likelihood method with default parameters and then visualized with iTOL (Letunic et al., 2007). The *Ach-CEN153* and *Ae-CEN153* sequences were aligned and visualized using DNAman V9.0.

### Supplementary Information

**Additional file 1:** **Supplementary Fig. 1.** Tree and fruit of *A. eriantha *‘Midao 31’. The average soluble solid content is 19.6 %, the average fruit weight is 76.2 g, the average acidity is 0.88%, and the average ASA content is 695.76 mg/100g. **Supplementary Fig. 2.** Squence alignment between MDHAPs and other genomes. **Supplementary Fig. 3. **KEGG(a) and GO(b) enrichment of newly predicted genes in MDHAPA. **Supplementary Fig. 4. **KEGG(a) and GO(b) enrichment of newly predicted genes in MDHAPB. **Supplementary Fig. 5. **K-mer spectrum analysis. The plots are colored to illustrate how many times of special K-mers from the hifi reads appearing in the assembly. **Supplementary Fig. 6. **BUSCO assessment of A.eriantha genome assemblies. **Supplementary Fig. 7. **GO(a) and KEGG(b) enrichment of ASES. **Supplementary Fig. 8. **Consistent and in consistent allele-specific expression (ASE) pattern across six sample. (a) Consistent ASEs. (b) inconsistent ASEs. The color bar represents log2(FC) values. FC indicates fold change of FPKM values between allele A and allele B. Red color suggests that expression in allele A is significantly higher than allele B and blue color means that expression in allele B is significantly higher than allele A. DAF means day after fruiting. **Supplementary Fig. 9. **Sequence alignment of *A. eriantha *and *A.chinensis *representative centromere (153bp) monomers.

**Additional file 2:** **Supplementary Table 1.** Summary of the data sequenced by multiple technologies. **Supplementary Table 2.** The remaining gaps in MDHAPA and MDHAPB. **Supplementary Table 3.** Comparison of repetitive elements between MDHAPA and MDHAPB. **Supplementary Table 4.** Statistics of genomic variation releated to new predicted genes between MDHAPs and White. **Supplementary Table 5.** Mapping rate of Illumina,HIC,HIFI,ONT reads for MDHAPA and MDHAPB. **Supplementary Table 6.** Statistics of genomic variation between MDHAPA and MDHAPB. **Supplementary Table 7.** Functional impact of the identified SNPs and InDels between MDHAPA and MDHAPB. **Supplementary Table 8.** The identified telomeres in MDHAPA. **Supplementary Table 9.** The identified telomeres in MDHAPB. **Supplementary Table 10.** The identified centromeres in MDHAPA and MDHAPB. **Supplementary Table 11.** New predicted genes in the centromere region of MDHAPA. **Supplementary Table 12.** New predicted genes in the centromere region of MDHAPB. **Supplementary Table 13.** The identified monomers in MDHAPA. **Supplementary Table 14.** The identified monomers in MDHAPB. **Supplementary Table 15.** The 10 RNA-seq reads used in this study.

## Data Availability

All data generated or analyzed during this study are included in this published article.

## Abbreviations

*A*. *eriantha*: *Actinidia eriantha*
*A*. *chinensis*: *Actinidia chinensis*
T2T: Telomere-to-telomere
GO: Gene Ontology
KEGG: Kyoto Encyclopedia of Genes and Genome
ASE: Allele-specific expression
