# Supplementary material for: Telomere-to-telomere and haplotype-resolved genome of the kiwifruit Actinidia eriantha
Source: Mol Hortic. 2023 Feb 17;3:4. doi: 10.1186/s43897-023-00052-5 (PMC10515003; doi:10.1186/s43897-023-00052-5)
Supplement: Supplementary file 1 — Additional file 1: Supplementary Fig. 1. Tree and fruit of A. eriantha ‘Midao 31’. The average soluble solid content is 19.6 %, the average fruit weight is 76.2 g, the average acidity is 0.88%, and the average ASA content is 695.76 mg/100g. Supplementary Fig. 2. Squence alignment between MDHAPs and other genomes. Supplementary Fig. 3. KEGG(a) and GO(b) enrichment of newly predicted genes in MDHAPA. Supplementary Fig. 4. KEGG(a) and GO(b) enrichment of newly predicted genes in MDHAPB. Supplementary Fig. 5. K-mer spectrum analysis. The plots are colored to illustrate how many times of special K-mers from the hifi reads appearing in the assembly. Supplementary Fig. 6. BUSCO assessment of A.eriantha genome assemblies. Supplementary Fig. 7. GO(a) and KEGG(b) enrichment of ASES. Supplementary Fig. 8. Consistent and in consistent allele-specific expression (ASE) pattern across six sample. (a) Consistent ASEs. (b) inconsistent ASEs. The color bar represents log2(FC) values. FC indicates fold change of FPKM values between allele A and allele B. Red color suggests that expression in allele A is significantly higher than allele B and blue color means that expression in allele B is significantly higher than allele A. DAF means day after fruiting. Supplementary Fig. 9. Sequence alignment of A. eriantha and A.chinensis representative centromere (153bp) monomers. [file 43897_2023_52_MOESM1_ESM.pdf]

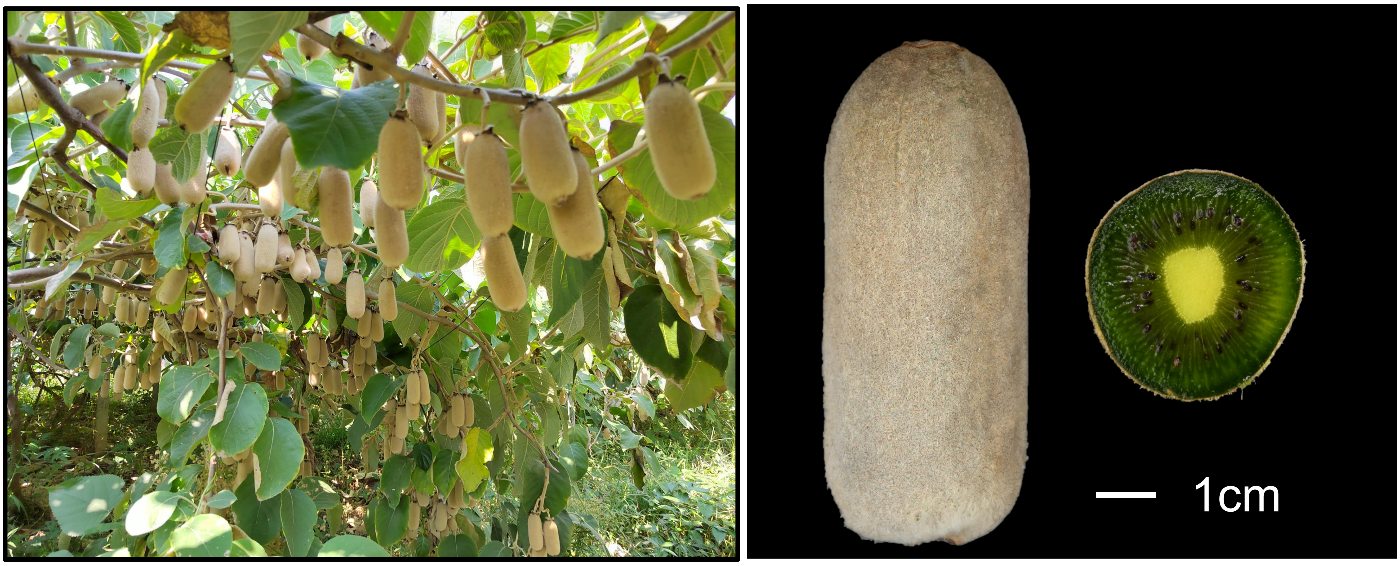

Supplementary Fig. 1 Tree and fruit of ***A. eriantha*** **'Midao 31'.** The average soluble solid content is 19.6 %, the average fruit weight is 76.2 g, the average acidity is 0.88%, and the average ASA content is 695.76 mg/100g.

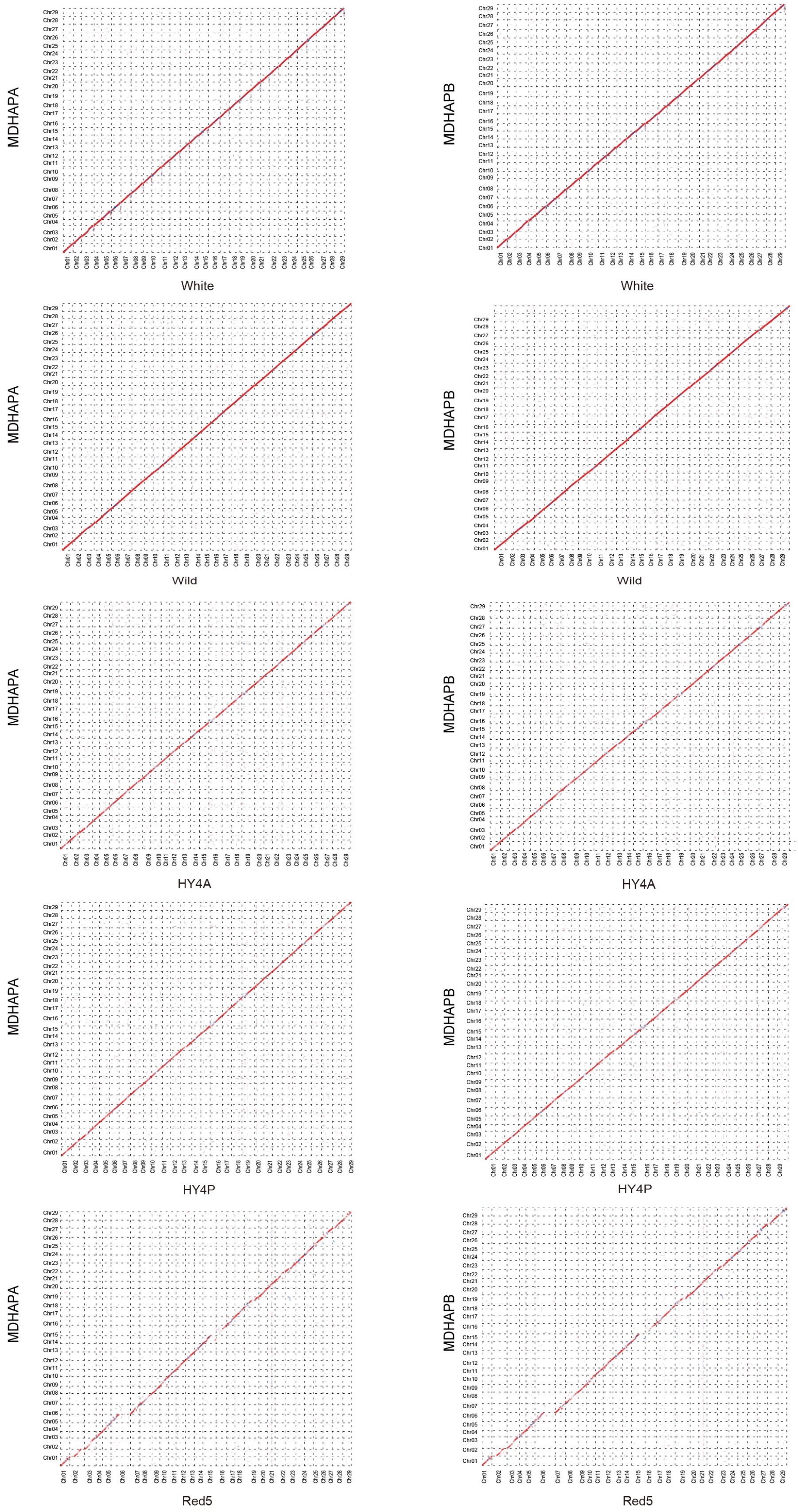

Supplementary Fig. 2 Squence alignment between MDHAPs and other genomes.

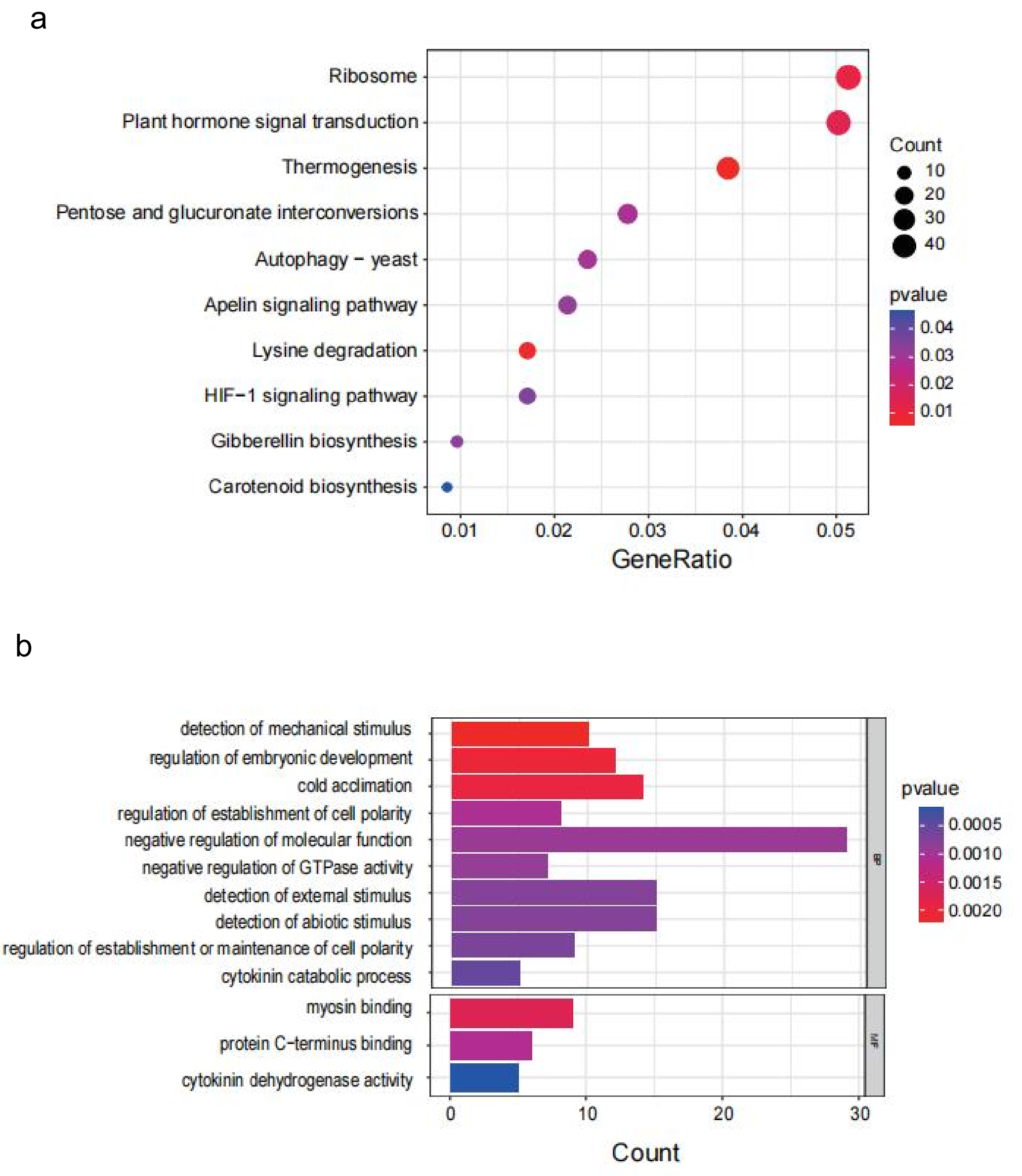

Supplementary Fig.3 KEGG(a) and GO(b) enrichment of newly predicted genes in MDHAPA.

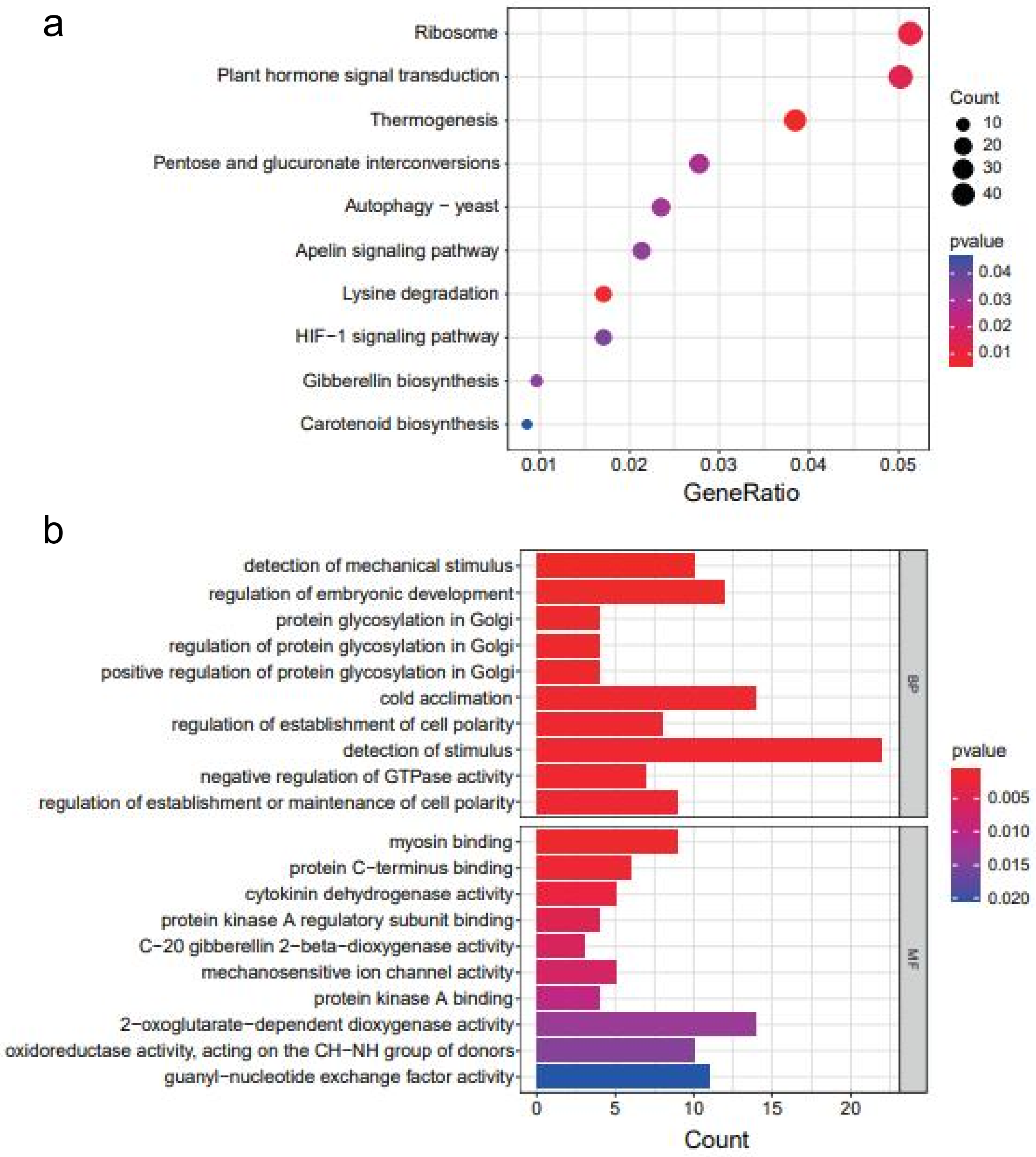

Supplementary Fig.4 KEGG(a) and GO(b) enrichment of newly predicted genes in MDHAPB.

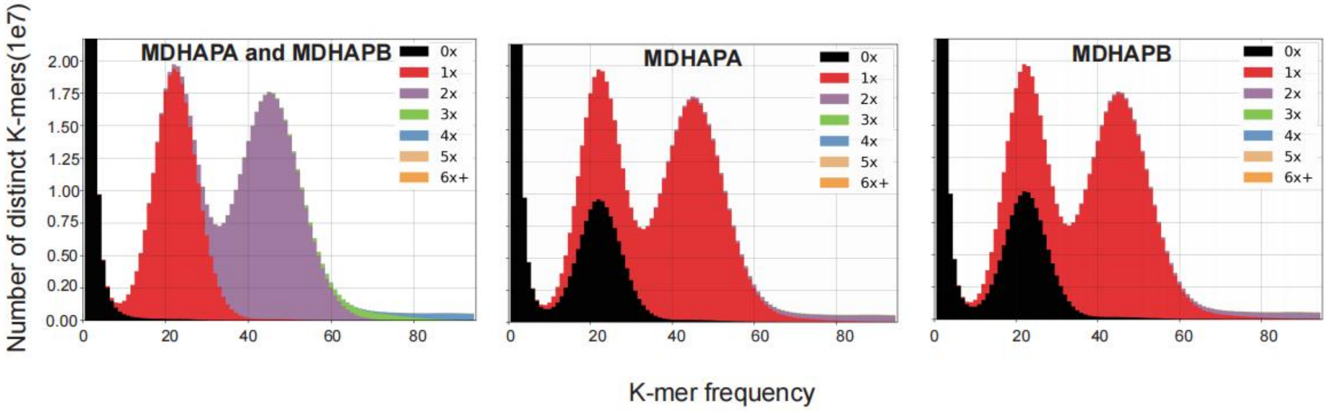

Supplementary Fig. 5 K-mer spectrum analysis. The plots are colored to illustrate how many times of special K-mers from the hifi reads appearing in the assembly.

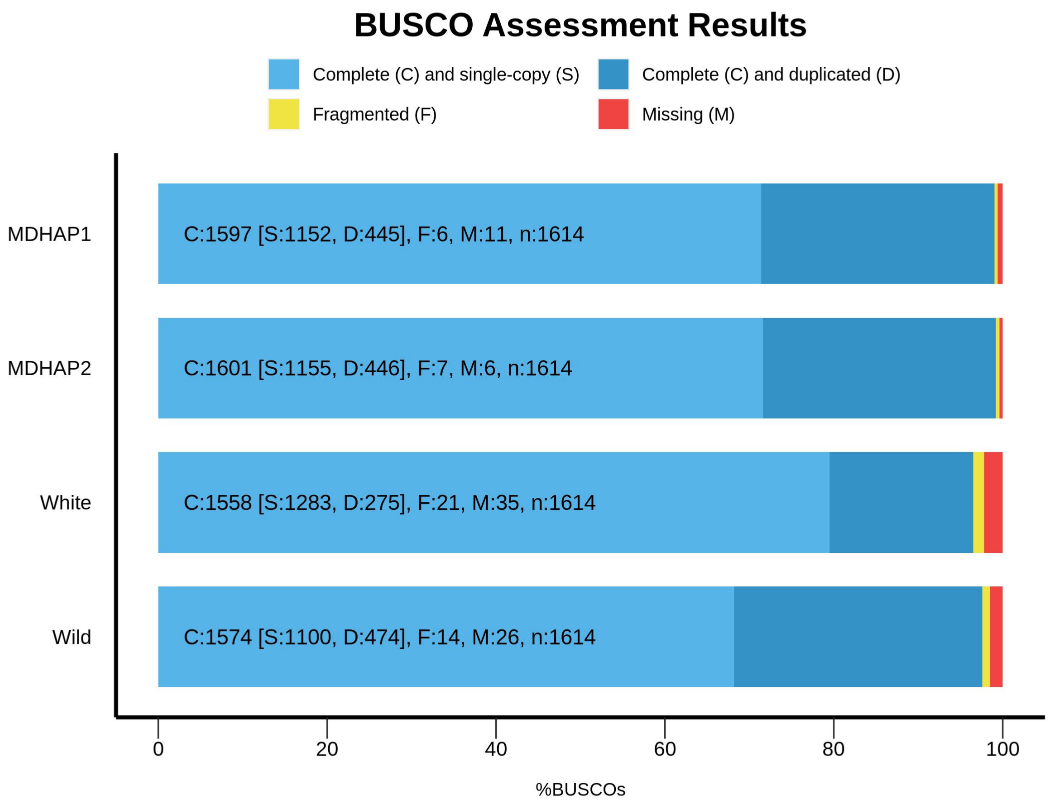

Supplementary Fig. 6 BUSCO assessment of A.eriantha genome assemblies.

a

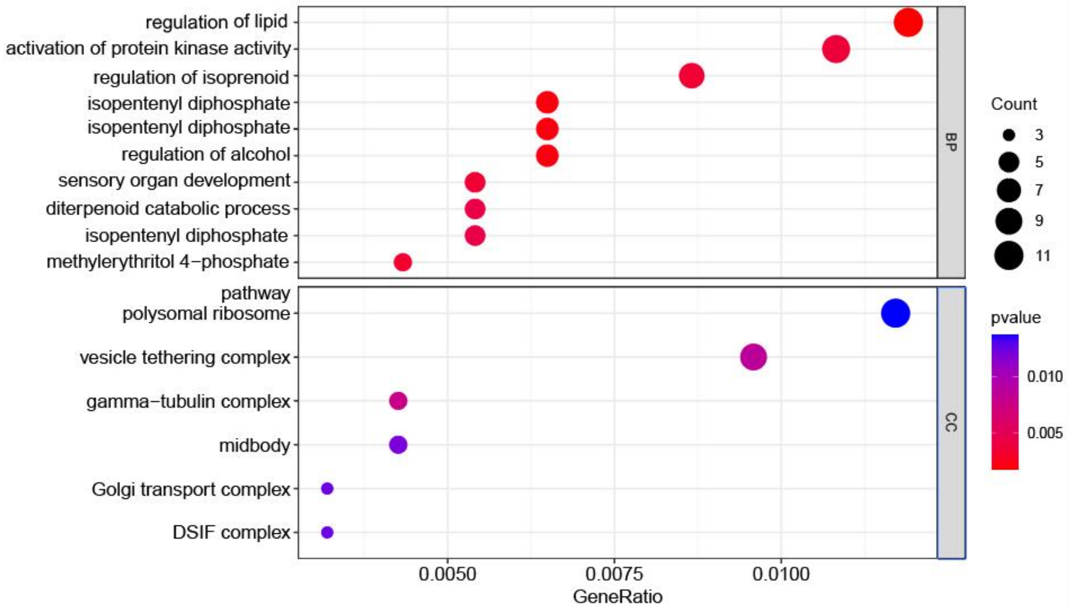

b

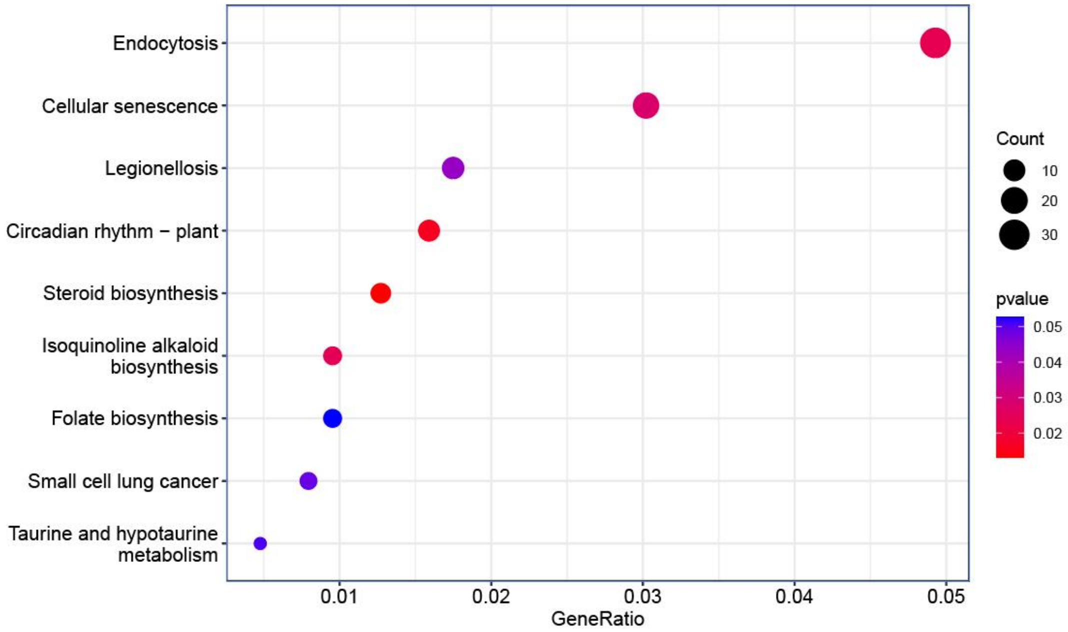

Supplementary Fig.7 GO(a) and KEGG(b) enrichment of ASES.

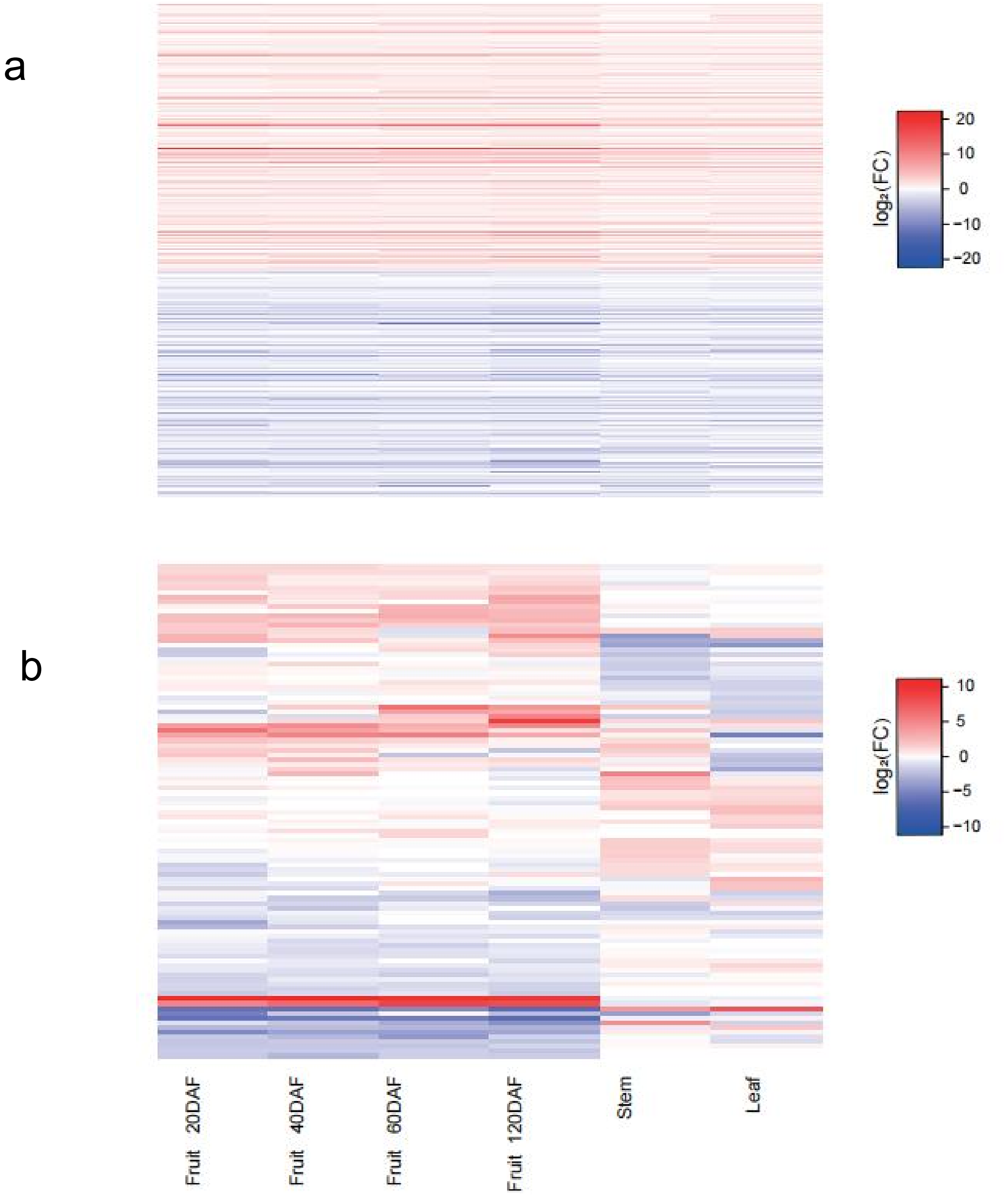

Supplementary Fig. 8 Consistent and in consistent allele-specific expression (ASE) pattern across six sample. (a) Consistent ASEs. (b) inconsistent ASEs. The color bar represents log_2(FC) values. FC indicates fold change of FPKM values between allele A and allele B. Red color suggests that expressionin allele A is significantly higher than allele B and blue color means that expression in allele B is significantly higher than allele A. DAF means day after fruiting.

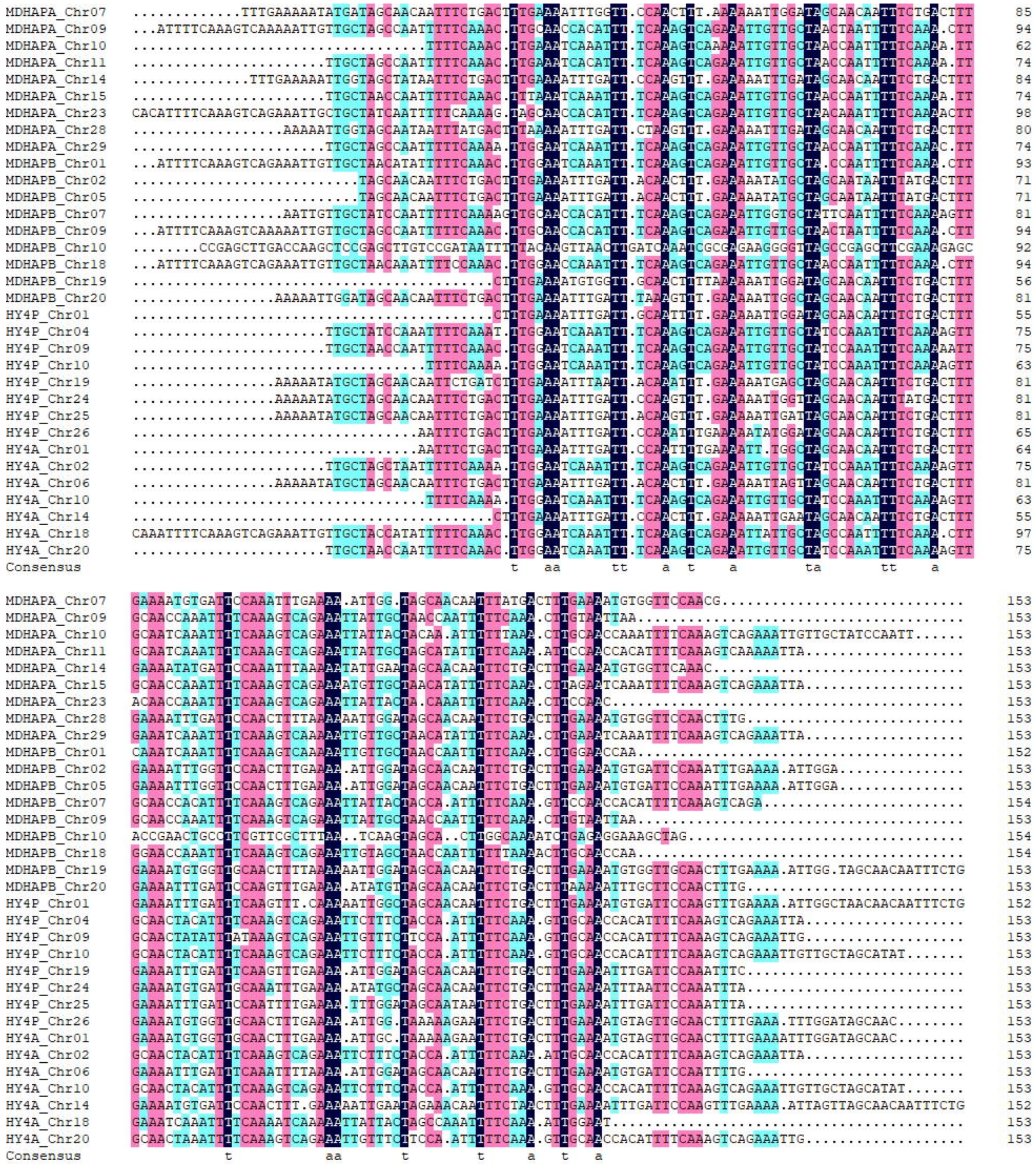

Supplementary Fig. 9 Sequence alignment of *A. eriantha* and *A.chinensis* representative centromere (153bp) monomers.
